# Role of Peripheral Immune Cells-Mediated Inflammation on the Process of Neurodegenerative Diseases

**DOI:** 10.3389/fimmu.2020.582825

**Published:** 2020-10-15

**Authors:** Qiuyu Yang, Guoqing Wang, Feng Zhang

**Affiliations:** Key Laboratory of Basic Pharmacology of Ministry of Education and Laboratory Animal Center and Joint International Research Laboratory of Ethnomedicine of Ministry of Education, Zunyi Medical University, Zunyi, China

**Keywords:** neurodegenerative diseases, peripheral immune cells, macrophage, dendritic cell, natural killer cell, T cell, B cell, monocyte

## Abstract

Neurodegenerative diseases are characterized by progressive loss of selectively vulnerable neuronal populations, which contrasts with selectively static loss of neurons due to toxic or metabolic disorders. The mechanisms underlying their progressive nature remain unknown. To date, a timely and well-controlled peripheral inflammatory reaction is verified to be essential for neurodegenerative diseases remission. The influence of peripheral inflammation on the central nervous system is closely related to immune cells activation in peripheral blood. The immune cells activation participated in the uncontrolled and prolonged inflammation that drives the chronic progression of neurodegenerative diseases. Thus, the dynamic modulation of this peripheral inflammatory reaction by interrupting the vicious cycle might become a disease-modifying therapeutic strategy for neurodegenerative diseases. This review focused on the role of peripheral immune cells on the pathological progression of neurodegenerative diseases.

## Introduction

Neurodegenerative disease is the progressive dysfunction and loss of neurons in the central nervous system (CNS), including Alzheimer's disease (AD), Parkinson's disease (PD) and Multiple Sclerosis (MS) (1). The mechanisms underlying their progressive nature remain unknown. To date, aging and immunity are closely associated with the pathogenesis of neurodegenerative diseases. Immunosenescence refers to the gradual deterioration of the immune system brought on by natural age advancement. It involves both the host's capacity to respond to infections and the development of long-term immune memory, which could accelerate the progression of neurodegenerative diseases (2).

Despite different triggering events, a common feature is brain inflammation (3). It is clear that neuroinflammation during compensatory period is beneficial, which help combat infections, promote tissue repair, remove necrotic cells, shape the brain during development and repair following damage. Upon decompensatory period, a vicious cycle of glial priming and release of pro-inflammatory factors promote neuronal damage (4). On the other hand, chronic inflammation, including chronic intestinal inflammation, diabetes, obesity, and systemic lupus erythema, could cause cognitive impairment, learning and memory deficits, and human depression (5, 6). Moreover, long-term use of non-steroidal anti-inflammatory drugs would suppress the peripheral immunity and reduce the incidence of PD by about 50% (7). In PD mice model, intraperitoneal lipopolysaccharide (LPS) injection combined with intravenous administration of two different recombinant α-synuclein (α-syn) pathogenic strains resulted in overactivation of microglia and further promoted the recruitment of leukocytes toward the brain and the spinal cord (8). Likewise, inhibiting migration of T cells or B cells into the brain rendered the CNS susceptible to devastating infections. However, the nature of peripheral immune cells in neurodegenerative diseases progression remains unclear. Thus, this review summarized the roles of peripheral immune cells on the pathological progression of neurodegenerative diseases.

## Roles of Peripheral Immune Cells on Neurodegenerative Diseases

### Mononuclear Phagocyte System

#### Monocyte

Monocyte is the largest type of white blood cell in the peripheral blood that could differentiate into macrophages or dendritic cells (DCs) (9). Monocyte triggers innate immune responses by regulating Toll-like receptors (TLRs), scavenger receptors, phagocytosis and complement-mediated responses. Recent studies revealed that gut dysbiosis, a primary element behind various gastrointestinal disorders, might augment LPS, pro-inflammatory factors and monocytes, thus leading to increased intestinal and blood brain barrier (BBB) permeability through microbiota-gut-brain axis. Correspondingly, accumulation of axonal damage, misfolded proteins and neuronal demyelination facilitates the pathogenesis of neurodegenerative disorders, such as AD, PD and MS (10).

In AD patients, a higher proportion of monocytes in the peripheral blood was discerned, whereas the interaction between monocytes and platelets in the blood was not altered. Besides, cathepsin D, a major lysosomal aspartic protease, was underexpressed in monocytes, causing the defective degradation of amyloid-β (Aβ) by monocytes (11). However, the sensitivity of monocytes toward Aβ peptides was decreased, indicating that there might be a critical link between the interaction of platelets and monocytes in AD (12).

Transcriptomics analysis showed that monocytes isolated from peripheral blood of PD patients conferred pro-inflammatory effects. The increase in the number of classical monocytes in PD blood and the decrease in the number of non-classical monocytes might result from the increased monocyte differentiation or increased migration from the bone marrow (13). In contrast, monocytes play an important role in repairing of the injured brain. For example, continuous low-dose injections of LPS in the periphery caused chronic inflammation and the tolerance of peripheral monocytes. Once CNS was stimulated again, dopaminergic neuronal damage was reduced (14). Of note, PD-associated gene DJ-1 deficiency attenuated monocyte infiltration into the damaged brain, which in turn led to delay in repairing of brain injury in mice (15). Furthermore, the chemotaxis and phagocytosis of aged monocytes were increased or decreased under different conditions. In neurodegeneration, an increase in the number of monocytes and functional changes observed in peripheral blood might be related to immunosenescence, but this change was more obvious in age-matched PD patients (16).

Currently, the blood monocyte counted in the early phase of MS was robustly associated with the clinical severity of MS, whereas the counts of the other blood cells were not related with MS severity (17). Moreover, various animal studies carried out that monocytes contributed to MS-associated neuroinflammation. While classically activated monocytes promoted inflammation, type II-activated monocytes could improve the progression of MS. Furtherly, antioxidant and anti-inflammatory alternatives inhibited monocyte secretion of pro-inflammatory cytokines, such as TNF-α, IL-6, and IL-1β, and also suppressed the phagocytosis of monocytes and thus slowed down the pathological process of MS (18).

#### Macrophage

In the inflammatory lesions, macrophages are the dominant cells. Macrophages in peripheral blood can cross BBB to secrete pro-inflammatory factors in brain to further determine the survival of neurons (19). Production of these inflammatory factors in brain is generally considered to be the primary mechanisms underlying the development of neuronal damage in response to chronic inflammation (20). Additionally, the renin-angiotensin system acts on macrophages via different signaling pathways. Angiotensin (Ang) II type 1 receptors (ATR) drive pro-inflammatory macrophage responses in neuroinflammation via regulation of chemokines. Interestingly, macrophages could secrete pro-inflammatory and anti-inflammatory factors due to the autoimmune actions of inflammation (21). In CNS, microglia are the resident macrophages and play vital functions for brain development and homeostasis. The phenotypic differentiation between microglia and peripheral macrophages is verified to be age-dependent. Peripheral macrophages might express several most commonly described microglia markers in some developmental stages or pathological conditions, particularly during chronic neuroinflammation (22). At present, blood-derived macrophages are thought to contribute to brain damage and repair in yet unidentified ways (23).

A number of studies demonstrated that defects of macrophages interfered with brain clearance of Aβ, including in Aβ phagocytosis and Aβ-induced apoptosis. Macrophages derived from peripheral blood in AD patients were found to possess ineffective phagocytosis of Aβ and low resistance to apoptosis by Aβ (24–26). Another evidences indicated that IL-34 could impair monocyte differentiation into macrophages and reduce their ability to uptake pathological forms of Aβ. Given the critical role of macrophage-mediated Aβ clearance in both murine models and patients with AD, IL-34 might be relevant to innate immune responses in AD (27). Besides, in clinical studies, the highly reactive compound, methylglyoxal, has been implicated in the development of AD and methylglyoxal might be produced by macrophages during sepsis and further fasten the pathological process of AD (28).

In addition, modulation of the secretion of anti-inflammatory factors by macrophages might be a reasonable way to control chronic inflammation and delay the progression of neurodegenerative diseases. In PD patients, niacin could reduce the expression level of GPR109a in macrophages and increase the secretion of anti-inflammatory factors by macrophages in blood, that thereby slowing the progression of PD (29, 30). On the other hand, intraperitoneal injection of 1-methyl-4-phenyl-1, 2, 3, 6-tetrahydropyridine (MPTP) in mice increased peripheral macrophages levels (31). Also, macrophages can activate PD-related genes, such as LRRK2, by pathogen- or sterile-induced endomembrane damage (32). Meanwhile, glial cell-line derived neurotrophic factor (GDNF) delivery mediated by macrophages from bone marrow was confirmed to improve neuroinflammation and inhibit dopaminergic neurodegeneration (33).

Macrophages are the predominant cell type in acute inflammatory brain lesions of MS, which can produce pro-inflammatory and toxic molecules and promote demyelination, although macrophages of peripheral origin were not normally present in the parenchyma of healthy CNS (34, 35). In detail, in response to experimental autoimmune encephalomyelitis (EAE, the induced variant of MS in animals, usually in mice) induction, they were recruited and infiltrated into the CNS and, together with residential microglia, contributed to the pathogenesis of MS (36). Moreover, IL-10 and IL-4 immunoreactivity were shown in active demyelinating lesions and the rim of chronic active lesions of human MS brain, with receptors for these cytokines highly expressed by macrophages in parenchymal and perivascular areas (37). Of note, myelin-laden macrophages expressing high levels of macrophages-associated CD163 and CD206 were discerned in MS patient lesions (38).

### Dendritic Cells (DCs)

DCs had various functions and were recognized as a translator for innate and adaptive immunity. They integrated signals from tissue infection or injury, migrated inflammatory sites and processed antigens to be presented to secondary lymphoid organs. Also, DCs provided a variety of soluble and surface-bound signals to help guide T cell differentiation (39). Generally, decreased number of DCs and decline in DCs functions are the key hallmarks of immunosenescence. Changes of DCs in neurodegenerative diseases patients thus closely resemble classical immunosenescence, and cannot be excluded that neurodegenerative diseases were just characterized by accelerated aging of the immune system.

Multiple studies demonstrated that during inflammation of CNS, activated DCs migrated to cervical lymph nodes, where DCs activated naive lymphocytes and then migrated to the site of inflammation (40). Although DCs were easily found in cerebrospinal fluid, they were not protected by BBB in the perivascular space. Additionally, the resident or infiltrating DCs exerted anti-inflammatory functions. Intranasal application of vesicular stomatitis virus (VSV) could cause acute infection of CNS. However, in DCs-deficient mice, the interferon-γ (IFN-γ) response induced by VSV in CNS was still intact. Therefore, it was still believed that inflammation and certain components of the adaptive primary anti-viral immune response in CNS were dependent on the peripheral DCs in the body (41).

The presence of DCs was still a matter of debate in AD. However, a surprising decline in the population of precursors of DCs in peripheral blood of AD patients with concomitant decline in blood myeloid DCs (MDC) was reported (42). Depletion of DCs by systemic injection of diphtheria toxin, which selectively targeted and eliminated bone-marrow-derived DCs, led to the increased levels of amyloid plaques in AD animal models. These findings suggested that the peripheral DCs were recruited in the brain and participated in the clearance of amyloid plaques (43). This phenomenon appeared mainly linked to AD progression and influenced by acetylcholinesterase inhibitors treatment (44). What's more, AD-afflicted hippocampi were also composed of more active DCs and fewer resting DCs than healthy people (45). Furthermore, the activation of DCs prior to the gradual loss of neighboring sensory neurons suggested an early involvement of immune cells in tau-associated pathology originating in CNS (46). Otherwise, studies on the ability of DCs to induce protective immunity to neurodegenerative diseases might have important implications for the development of novel strategies for prophylactic and therapeutic immunizations against microbial pathogens. Vaccination of DCs sensitized to Aβ with a T cell epitope mutation generated antibody responses in AD mice (43). These above findings indicated that DCs might play a critical role in the pathogenesis of AD.

In PD patients, the functional changes of DCs in the peripheral system were well-studied. For example, changes in circulating MDC and lymphocyte-like DCs (LDC) on the serum of PD patients were detected. MDC migrated in inflamed tissues and lymph nodes and recognized pathogens. LDC differentiated into typical DCs and mainly produced interferon (47). Recently, it was suggested that peripheral DCs in PD mice model might enter the brain and located in the choroid plexus or meninges and then react with various antigens to promote neuroinflammatory processes (48). In addition, tolerogenic bone marrow-derived DCs induced neuroprotective regulatory T cells (Tregs) in MPTP-induced PD mice model (49). Surprisingly, dendritic cell factor 1 (DCF1), a membrane protein that plays important roles on nerve development in mouse, could prevent α-syn-induced dopaminergic neuron loss by aggregating α-syn in the dorsomedial region of Drosophila (50).

Migration of DCs to CNS was also a critical event in the pathogenesis of MS. Upon the trafficking of human DCs subsets, circulating MDC and LDC in the blood of MS patients were exhibited, although the exact role of LDC in the pathogenesis of MS remained controversial. Importantly, LDC activation was enhanced in MS and the costimulatory molecules, such as OX40-L, HLA-DR, and CD86 expressed on LDC, could mediate a protective response against the viral trigger of autoimmunity (51).

### Natural Killer (NK) Cell

NK cells were active members of the innate immunity response system and act as a first-line defense or as non-specific effectors (52). It is found that the number of NK cells increases with age, but changes in NK cells function are less clear. The overwhelming evidence indicated the depressed NK cells function in old individuals. The clinical manifestations attributed to immunosenescence could also be the result of age-dependent alterations in NK cells number and function (53).

At present, the roles of NK cells on the pathogenesis of neurodegenerative diseases were still unilluminated. For example, changes of peripheral NK cells functions in patients with AD and healthy elderly people were shown, while NK cells killing activity and degranulation (CD107 expression) were unchanged. The underlying mechanisms were unrevealed (54). Moreover, there was no significant difference in the frequency of NK cells in AD patients, but increased spontaneous release of IFN-γ and TNF-α from NK cells was exhibited compared to healthy subjects (55). In addition, compared with healthy subjects, circular RNA changes in AD patients, and these RNAs were related to NK cell-mediated cytotoxicity (56). In PD patients, the percentage of NK cells was increased, while the activity of NK cells was not changed (57). Amounts of evidence showed that NK cell levels might be positively associated with the severity of PD (58). Additionally, NK cells modulated α-syn pathology and motor symptoms in α-syn transgenic mice model (59). Besides, the level of NK cells in peripheral blood of MS patients was increased (60). However, the roles of NK cells on MS were still controversial with studies reporting both protective and damaging roles in MS animal models (61). Collectively, the potential role of NK cells on neurodegenerative diseases warrants deep illustration.

### T Cell

**Figure 1:**
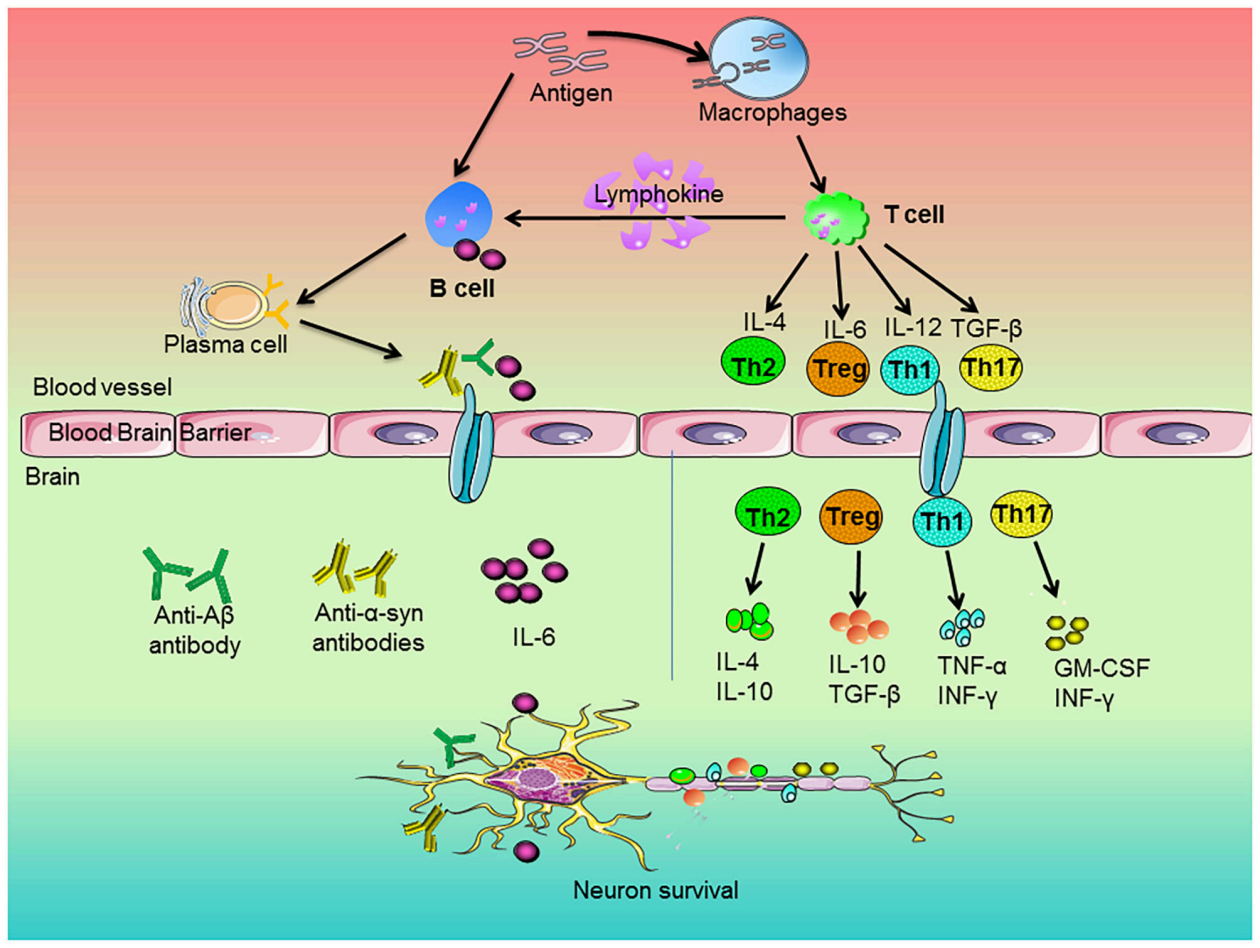
Roles of peripheral immune cells on the pathogenesis of neurodegenerative diseases. The antigen stimulated the immune response of macrophages and B cells. Macrophages engulfed antigens and presented them to T cells. The cytokines of IL-4, IL-6, IL-12, and TGF-β released by T cells regulated the development of Th2, Treg, Th1, and Th17 cells, respectively. Then, these cells secreted anti-inflammatory or pro-inflammatory factors to regulate neuronal survival. In addition, antigens could directly stimulate B cells. Upon activation, B cells produced pro-inflammatory factors, which entered the brain along blood vessels and participated in neurodegeneration. On the other hand, activated T cells secreted lymphokines to activate B cells and activated B cells could proliferate and differentiate into plasma cells. Subsequently, the plasma cells-produced cytokines and antibodies, such as anti-Aβ or anti-α-synuclein antibodies, went across blood brain barrier and entered the brain and thus attenuated neurons degeneration.

T cells could penetrate into CNS after being damaged, which affected the activation of glial cells and the degeneration of neurons (62). The activation of T cells depends on the antigen-presenting cells (APCs). The blood cerebrospinal fluid lacks APCs. APCs are recruited in cerebrospinal fluid of neurodegenerative diseases. Then, APCs present antigens peptides through major histocompatibility complex (MHC) class II molecules to further activate infiltrating T cells and the process of their surface T cell receptor (TCR) bound these presented antigen peptides. The TCR-MHC interaction induces the production of CD4^+^ T cells and CD8^+^ T cells (63). Undifferentiated CD4^+^ T cells activated by APCs differentiated into different functional phenotypes under different mediators. CD4^+^ T cells tended to become T-helper 1 (Th1) phenotype and Th17 inflammatory phenotype, which was closely related to neuroinflammation and neuronal damage (64). On contrast, CD4^+^ T cells could differentiate into the functional Th2 and Tregs. These phenotypes played a fundamental role on inhibitory effects on the inflammatory functions of T cells and the reduction of neuroinflammation (65, 66). Recently, as shown in Figure 1, studies indicated that Th1 and Th17 cells secreted pro-inflammatory factors, such as TNF-α and INF-γ, to induce microglia activation and the subsequent release of other pro-inflammatory factors (67), whereas Th2 cells and Tregs produced IL-4 and IL-10 to promote the shift of microglia from pro-inflammatory to anti-inflammatory phenotypes (68).

Fundamentally, brain aging drives systemic aging of whole body, including aging-associated changes of immune system. In turn, the immune system aging, particularly immunosenescence and T cell aging, initiated by thymic involution that are sources of chronic inflammation in the elderly (termed inflammation), potentially elicits brain aging and neurodegeneration in a reciprocal manner (69). In detail, mounting evidence have also emphasized that peripheral T lymphocytes played an essential role on the process of neuroinflammation in AD pathogenesis. Furthermore, role of Aβ-specific T cells in AD was bidirectional that they might act in either protective or damaging properties (70). On the other hand, T cells promoted hippocampal neurogenesis in AD mice model and T cells deficiency restricted neuronal regeneration in the hippocampus. The mechanisms underlying the promotion of neuronal regeneration by T cells were mediated by an up-regulated expression of peripheral T cells and brain microglial neurotrophic factors release (71, 72). Recently, a T-cell population called CD8^+^ effector memory CD45RA^+^ T cells (T_EMRA_ cells) was identified to be closely associated with AD. In cohort of 29 AD patients and 35 healthy controls, CD8^+^ T_EMRA_ cells correlated with cognitive dysfunctions, and the presence of these T cells could predict the disease severity with 80% accuracy (73, 74).

In PD patients, T cell levels were down-regulated in peripheral blood (75). Cognitive impairment was associated with higher number of circulating lymphocytes and dysregulation of Tregs compartment (76). It was interesting to note that levels of IL-1β, TNF-α, IL-2 and peripheral blood lymphocytes in the serum and cerebrospinal fluid of patients with PD were quite high, suggesting CD4^+^ and CD8^+^ T lymphocytes were involved in PD progression (77). In addition, Tregs might exert immunoregulatory functions through the interaction of the peripheral and central immune systems. Indeed, it has been demonstrated that in MPTP-induced PD mouse model, Tregs conferred neuroprotection against dopaminergic neuronal loss in the substantia nigra (78). Similarly, Tregs functions were apparently decreased in the periphery of 6-OHDA-induced PD rat model, in which T cell infiltration occurred when neuronal loss in substantia nigra reached 80% (79). Moreover, a relationship between α-syn-specific T cells and PD emerged that the presence of these T cells might be a feature of preclinical and early motor PD (80).

MS was traditionally recognized as a predominantly T cell-mediated autoimmune disease (81). First, the MHC class II allele was known for several decades to be the strongest genetic risk factor for MS. MHC class II proteins were expressed on APCs and required for antigen presentation to CD4^+^ T cells (82). Second, the elevated expression of multiple TGF-β-targeting miRNAs in naive CD4^+^ T cells of patients with MS impaired TGF-β signaling, and dampened Tregs development, thereby enhancing the susceptibility to developing MS (83). Otherwise, Th cell-induced expression of IL-26 was up-regulated in the blood and cerebrospinal fluid of patients with MS. In EAE, IL-26 reduced disease severity and pro-inflammatory lymphocyte infiltration into CNS, while increasing infiltration of Tregs (84, 85). To date, most studies of the human microbiome have focused on the roles of gut microbiota on MS. Gut microbiota regulates T cell functions throughout whole body. Microbiota transplants from MS patients into germ-free mice resulted in more severe symptoms of EAE and the decreased proportions of IL-10^+^ Tregs (86). On the other hand, higher Th1 and Th17 proportion in MS patients was closly associated with more frequent relapse and more severe clinical disability (87).

### B Cell

B cells are involved in adaptive immunity and considered to be an important component to participate in the pathological process of neurodegenerative diseases (95). Besides, as shown in Figure 1, B cells performed various functions, including the antigen presentation to T cells, production of pro-inflammatory factors and secretion of anti-inflammatory cytokines (96). In detail, auto-reactive B cells played a pivotal role in autoimmune neurological disorders. B cells released cytokines to promote inflammatory responses of IL-6 and TNF-α and granulocyte-macrophage colony-stimulating factor (GM-CSF) to promote the differentiation of pro-inflammatory factors (97). On the other hand, B cells could secrete IL-10 and IL-35 to exert anti-inflammatory effects.

B cell population has been shown to decline due to age, contributing substantially to immunosenescence (98). B cell immunosenescence induces lower antibody specificity. Also, the antibody specificity is altered by aging. The impairments affecting B cells during aging are reduction of B cell number and decreased sensitivity to antigens. Thus, the senescence of B cells affects the pathological process of neurodegenerative diseases. For example, the decreased levels of peripheral B cell subsets were detected in AD patients, which might be associated with the genetic changes in these cells (88). Furthermore, differences of levels of anti-Aβ antibodies in serum or cerebrospinal fluid between AD patients and healthy controls were indicated. These inconsistencies might be related to the increased binding of anti-Aβ antibodies to Aβ in AD patients (89, 90). There is growing evidence that a reduced Aβ pathology was indicated in an amyloid precursor protein (APP) transgenic mouse model of AD lacking functional B cell. Overall, these results demonstrated an essential role Of B cells on cerebral Aβ pathology.

Additionally, there are several types of receptors on the surface of B cell membranes, which express IgM, IgD, IgG, IgA, and IgE. These immunoglobulins were important characteristic markers for B cells and bound to the corresponding receptors on immune cells to present different functions. In PD patients, B cells were not detected in the brains, while IgG precipitates were found in dopaminergic neurons and IgG coated in Lewy bodies (91). Although the exact specificity of the transferred IgG antibody was not defined, previous studies confirmed that in passive transfer experiments, a large amount of IgG derived from PD patients caused the gradual loss of selective dopaminergic neurons. Importantly, detection of cell levels in the serum of PD patients demonstrated that the decrease in CD4^+^ Th cells and CD19^+^ B cells was worsened with the increased clinical severity (99–101). On the other hand, through interrogating peripheral IgG^+^ memory B cells from PD patients for reactivity to α-syn, naturally occurring antibodies derived from PD patients suppressed α-syn seeding *in vitro* and recognized Lewy pathology. This finding suggests that the memory B cell repertoire of PD patients might represent a potential source for biomarkers and therapies (92).

What's more, B cells could be discerned in CNS lesions in early to late stages of MS and most B cells were confined to the perivascular space (93). B cells in MS were verified to display a pro-inflammatory cytokine profile. Furthermore, in human cytomegalovirus (HCMV)-encoded antigens in patients with MS, B cells from HCMV(–) MS patients induced an enhanced pro-inflammatory profile compared to HCMV(+) MS cases, suggesting that persistent HCMV infection might reduce the inflammatory responses of B cells in MS (102). Besides, injection of TNF^+^ IFN-γ viral vectors elicited extensive B cells and macrophages infiltration in the meninges. These results implied that B cells could activate TNF signaling pathways in cortical cells leading to neuronal death and subpial demyelination and thus contribute to clinical progression of MS (94). Furthermore, B cell activation inhibitors inhibited the release of pro-inflammatory factors and impaired the capacity of B cells to act as APCs for the development of encephalitogenic T cells, resulting in selectively interfering with MS (103). In addition, anti-CD20-mediated B cell depletion effectively reduced acute MS flares. Currently, all approved MS disease-modifying therapies altered the frequency, phenotype or homing of B cells in one way or another. The importance of this action has been enhanced by the successful development and clinical testing of B cell-depleting monoclonal antibodies targeting the CD20 surface antigen (104). In addition, peripheral CD19^+^ B cells counts and infusion intervals were verified as a surrogate for long-term B cell depleting therapy in MS (105). Together, B cell-directed therapy in MS could be possibly advanced by integrating the emerging information on B cell regulation in MS into future therapeutic avenues.

## Conclusion

At present, as shown in Table 1, our understanding of the interaction between peripheral inflammatory mechanisms and neurodegenerative diseases and mutual regulation progressed greatly over decades. Peripheral immune cells are essential but not sufficient to cause neurodegenerative diseases. Additional triggers are clearly necessary for disease onset. Although enormous progress on the etiology of neurodegenerative diseases has been made, further critical investigation is warranted. The dynamic modulation of these peripheral inflammatory reactions by targeting peripheral immune cells might become a disease-modifying therapeutic strategy for neurodegenerative diseases.

**Table 1:** Effects of peripheral immune cells on the pathogenesis of neurodegenerative diseases.

	**Alzheimer's disease (AD)**	**Parkinson's disease (PD)**	**Multiple Sclerosis (MS)**
Monocyte	• A higher proportion of monocytes in the peripheral blood (11) • The sensitivity of monocytes toward Aβ peptides decreased (12)	• Not only exerted pro-inflammatory effects but also participated in repair of injured brain (14) • The number and function of monocytes increased in age-matched PD patients (16)	• The counts of blood monocytes associated with the clinical severity of MS (17) • Contributed to MS-associated neuroinflammation (18)
Macrophage	• Increased peripheral macrophages in AD mice (26) • Mediated the clearance and degradation of Aβ (24, 25)	• Increased peripheral macrophages in PD mice (31) • Produced pro-inflammatory and anti-inflammatory factors (30) • Activated LRRK2 after being stimulated by pathogens (32)	• Main cell type involved in MS (34) • Produced pro-inflammatory factors and promoted demyelination (35) • Infiltrating macrophages and microglia promoted the pathogenesis of MS (36)
Dendritic Cell (DC)	• Vaccination of DCs sensitized to Aβ generated antibody responses (43)	• Tolerogenic bone marrow-derived DCs induced neuroprotective regulatory T cells (Tregs) (49)	• Circulating myeloid DCs (MDC) and lymphocyte-like DCs (LDC) in the blood of MS patients exhibited • LDC activation enhanced in MS and the costimulatory molecules expressed on LDC, mediated a protective response against the viral trigger of autoimmunity
Natural Killer (NK) Cell	• Increased spontaneous release of IFN-γ and TNF-α from NK cells (55)	• NK cell levels positively associated with the severity of PD (59)Modulated α-synuclein pathology (59)	• The level of NK cells in peripheral blood of MS patients increased (60) • Conferred both protective and damaging roles in MS (61)
T Cell	• Might act in either protective or damaging properties (70) • Promoted hippocampal neurogenesis in AD mice (71, 72)	• T cell levels down-regulated in peripheral blood (75) • Cognitive impairment associated with higher number of circulating lymphocytes and dysregulation of Tregs compartment (76) • Tregs might exert immunoregulatory functions through the interaction of the peripheral and central immune systems (78, 79)	• MS traditionally recognized as a predominantly T cell-mediated autoimmune disease (81). • Higher Th1 and Th17 proportion in MS patients closely associated with more frequent relapse and more severe clinical disability (87)
B Cell	• Decreased levels of peripheral B cell subsets detected in AD patients (88) • Played an essential role on cerebral Aβ pathology (89, 90)	• Not detected in the brain, while IgG precipitates found in dopaminergic neurons and IgG coated in Lewy bodies (91) • Memory B cell repertoire of PD patients might represent a potential source for biomarkers and therapies (92)	• Were discerned in CNS lesions in early to late stages of MS (93) • Involved in neuroinflammation of cortical cells, leading to neuronal death and subpial demyelination and thus contributing to clinical progression of MS (94)

## Author Contributions

All authors read, revised, and approved the final manuscript.

## Conflict of Interest

The authors declare that the research was conducted in the absence of any commercial or financial relationships that could be construed as a potential conflict of interest.
